# American Medical Association

**Published:** 1860-08

**Authors:** 


### EDITORIAL AND MISCELLANEOUS.

### American Medical Association.

We were in attendance upon the late meeting of the American Medical Association, which convened at New Haven, Connecticut, on the 5th of June, and of course it would be very naturally expected that we should make some reference to its proceedings.

We take pleasure in testifying to the decided advance apparent in the scientific contributions to the body, and have no doubt that we shall have a valuable volume of "*Transactions*," made up of papers that have really no more connection with the *deliberations* of the Association than if they had never been presented, and which could just as readily have been published through the almost innumerable Medical Journals of the land, and in this way have been much more generally diffused. The most prominent point of remark, however, which occurred to us in connection with this meeting of the Association, *was the almost entire absence of Southern medical men*, thus constituting it essentially a *sectional* body. The truth is, as we have long since stated, the mass of the medical profession in the South feel no interest, whatever, in the perpetuation of an organization which is composed, to a very large extent, of elements with which they have no community of thought or interest, and which may be regarded as in no small degree decidedly inimical.

Thus, Southern representation in this so-called American Medical Association, has gradually diminished, until, without any such formal division as has taken place in the majority of the churches of the land upon sectional grounds, we find in the fact that there were only about thirty Southern men in a body containing in the aggregate at least 500, a most striking example of a real separation in connection with a mere nominal Association. If the occasion should require it, we are fully prepared to discuss the right and propriety of the claims for this body to regulate medical matters throughout the United States, set up by certain interested parties, who desire to use the Association for the advancement of their own selfish ends. We, however, forbear for the present, with the admission that so far as the citizens of New Haven are concerned, they deserve great credit for the hospitality extended to the members of the Association; and, notwithstanding the hot-bed of fanaticism in which we found ourselves, we cheerfully accord to them a refinement and cultivation rarely found anywhere.

